# A Limited Structural Modification Results in a Significantly More Efficacious Diazachrysene-Based Filovirus Inhibitor

**DOI:** 10.3390/v4081279

**Published:** 2012-08-15

**Authors:** Života Selaković, Dejan Opsenica, Brett Eaton, Cary Retterer, Sina Bavari, James C. Burnett, Bogdan A. Šolaja, Rekha G. Panchal

**Affiliations:** 1 University of Belgrade, Studentski trg 16, P.O. Box 51, Belgrade 11158, Serbia; Email: zivota.selakovic@gmail.com; 2 Institute of Chemistry, Technology, and Metallurgy, University of Belgrade, Belgrade 11000, Serbia; Email: dopsen@chem.bg.ac.rs; 3 United States Army Medical Research Institute of Institute of Infectious Diseases, Fort Detrick, 1425 Porter Street, Frederick, MD 21702, USA; Email: brett.eaton@amedd.army.mil (B.E.); cary.retterer@amedd.army.mil (C.R.); sina.bavari@us.arm.mil (S.B.); 4 SAIC-Frederick, Inc., Frederick National Laboratory for Cancer Research, P.O. Box B, Frederick, MD 21702, USA

**Keywords:** filovirus, Ebola virus, Marburg virus, antiviral, diazachrysene, inhibitory efficacy, toxicity, small molecule

## Abstract

Ebola (EBOV) and Marburg (MARV) filoviruses are highly infectious pathogens causing deadly hemorrhagic fever in humans and non-human primates. Promising vaccine candidates providing immunity against filoviruses have been reported. However, the sporadic nature and swift progression of filovirus disease underlines the need for the development of small molecule therapeutics providing immediate antiviral effects. Herein we describe a brief structural exploration of two previously reported diazachrysene (DAAC)-based EBOV inhibitors. Specifically, three analogs were prepared to examine how slight substituent modifications would affect inhibitory efficacy and inhibitor-mediated toxicity during not only EBOV, but also MARV cellular infection. Of the three analogs, one was highly efficacious, providing IC_50_ values of 0.696 µM ± 0.13 µM and 2.76 µM ± 0.21 µM against EBOV and MARV infection, respectively, with little or no associated cellular toxicity. Overall, the structure-activity and structure-toxicity results from this study provide a framework for the future development of DAAC-based filovirus inhibitors that will be both active and non-toxic *in vivo*.

## 1. Introduction

The Ebola (EBOV) and Marburg (MARV) filoviruses (order *Mononegavirales*, family *Filoviridae*) are non-segmented, single-stranded negative sense RNA viruses that cause severe hemorrhagic fever in humans and non-human primates [1]. Filoviruses are lethal and highly infectious, and therefore are classified as a Category A Bioterrorism Agents by the United States (US) Centers for Disease Control and Prevention [2]. For example, periodic filovirus disease in Africa results in high case fatalities [3], and as the infection proceeds at a rapid rate, there is little opportunity for developing acquired immunity.

There are currently no approved therapeutics or vaccines available to treat filovirus infections, although several promising vaccine candidates have been found to protect non-human primates [4,5,6]. However, the timeframe required for vaccine-acquired immunity (at least one month), *versus* the sporadic nature and swift progression of filovirus diseases, underlines the need for the development of small molecule countermeasures that will provide immediate therapeutic relief.

The development of therapeutics to treat filovirus infections has targeted the pathogen’s proteins, as well as host targets and pathways. In particular, antisense phosphorodiamidate morpholino oligomers designed to interfere with the translation of specific filovirus target genes have protected non-human primates against infection [7,8,9,10], while a chemical-genetic screening approach has identified several small molecule inhibitors providing anti-filovirus activity during cellular infection (and in some cases also *in vivo* protection) [11]. Additional examples of small molecule inhibitors include those with mechanisms of action that are currently unclear [12], as well as those that inhibit filovirus entry into host cells [12].

In 2009, Aman *et al*., reported a diazachrysene (DAAC)-based compound that provided *in vivo* protection against EBOV infection (in a murine model) when evaluated within either a prophylactic or therapeutic setting [13]. In the same study they also found that, in cell-based assays, the examined DAAC-based compound inhibited viral replication in divergent virus families [13]. Based on the results from the cellular assays, it was postulated that the antiviral mechanism of action of the compound involves targeting a conserved host pathway/mechanism [13]. Recently, we examined the anti-EBOV activities of thirteen diazachrysene (DAAC)-based congeners, and found that several provide varying degrees of cellular protection against EBOV infection [14]. Since that time, we have conducted a brief structural exploration of the DAAC chemotype in an attempt to gain a better understanding of the chemical features necessary for generating more efficacious inhibitors of both EBOV and MARV. In this communication, we report the anti-filovirus efficacies and cellular toxicities of three new DAAC analogs. Furthermore, for the most efficacious analog, we also show that the salt (cationic/ionized) form of the inhibitor is more efficacious than its respective basic (non-salt/unionized) form.

## 2. Results and Discussion

### 2.1. DAAC Analogs

**Figure 1:**
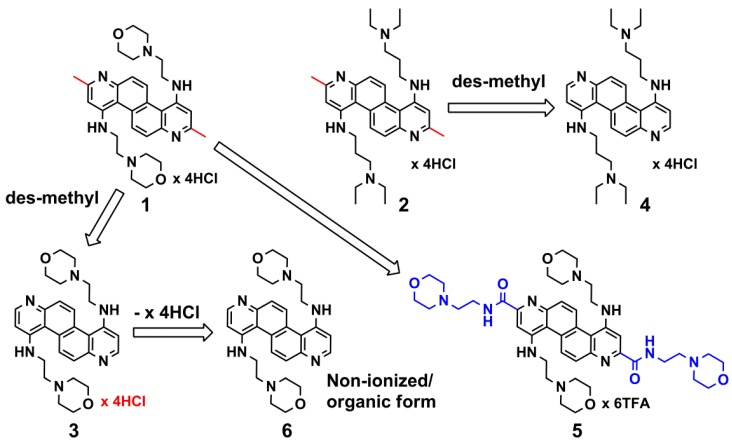
The chemical structures of compounds **1**–**6**. The bis-2,8-methyl substituents of parent compounds **1** and **2** are depicted in red. The term ‘des-methyl’ indicates the removal of these substituents to provide analogs **3** and **4**, respectively. For analog **5**, blue indicates the bis-2,8-amide-ethylmorpholino groups replacing the bis-2,8-methyl substituents of **1**.

Previously reported DAAC-based inhibitors of EBOV cellular infection all possess bis-2,8-methyl substituents and bis-4,10-alkylamino substituents [13,14]. Therefore, we selected two of the previously examined inhibitors for a brief ‘structural modification’ survey. The compounds included: **1** (Figure 1), which provided only 53% EBOV inhibition (at 20 µM), and was non-toxic to host cells; and **2** (Figure 1), which provided only 13% EBOV inhibition (at 20 µM), but was toxic to host cells. The rationale for choosing the two compounds: any structural modifications that might modify the efficacies and/or reduce the toxicities of either **1** or **2** during either EBOV and/or MARV infection would provide pronounced structure-activity data to be leveraged during the future syntheses of DAAC-based candidates for *in vivo* testing.

As indicated above, the structural modifications presented herein included a brief survey. Specifically, analogs **3** and **4** were synthesized to explore the impact on cellular efficacy and toxicity resulting from the removal of the bis-2,8-methyl substituents of **1** and **2** (Figure 1), and analog **5** was synthesized to examine the impact on cellular efficacy and toxicity resulting from replacing the bis-2,8-methyl substituents of **1** with bis-2,8-amide-ethylmorpholino groups (Figure 1). Furthermore, as **3** was found to be the most efficacious of the three analogs (*vide infra*), the basic (non-salt/unionized) form of the compound, analog **6** (Figure 1), was also examined to compare the effects, if any, of ionization state on inhibitory efficacy.

### 2.2. Anti-Filovius Activity

To initially gauge cellular efficacy and toxicity during EBOV and MARV infection, analogs **3**–**5** were screened at 20 µM. Specifically, HeLa cells were pretreated for 2 hrs with 20 µM of either **3**, **4**, or **5**, and subsequently infected with either three multiplicity of infection (MOI) EBOV Zaire-95 isolate (referred to as EBOV for the remainder of the text) or five MOI MARV Ci67 isolate (referred to as MARV for the remainder of the text). The preliminary results indicated that **3** and **4** provided 100% cellular protection during both EBOV and MARV infection, while **5** provided approximately 70–80% cellular protection during EBOV infection and 20–30% cellular protection during MARV infection. Toxicity analyses (based on cell number) indicated little or no cellular toxicity associated with **3**, while **4** caused 80–90% reduction in cell number in both assays. Compound **5** also showed cellular toxicity ranging from 30–40% in the EBOV assay and 20–30% in the MARV assay. Based on the cellular toxicity of compound **4**, it was eliminated as a candidate for further evaluation.

#### 2.2.1. Dose-Response Studies: Inhibitors **3** and **5**

**Figure 2:**
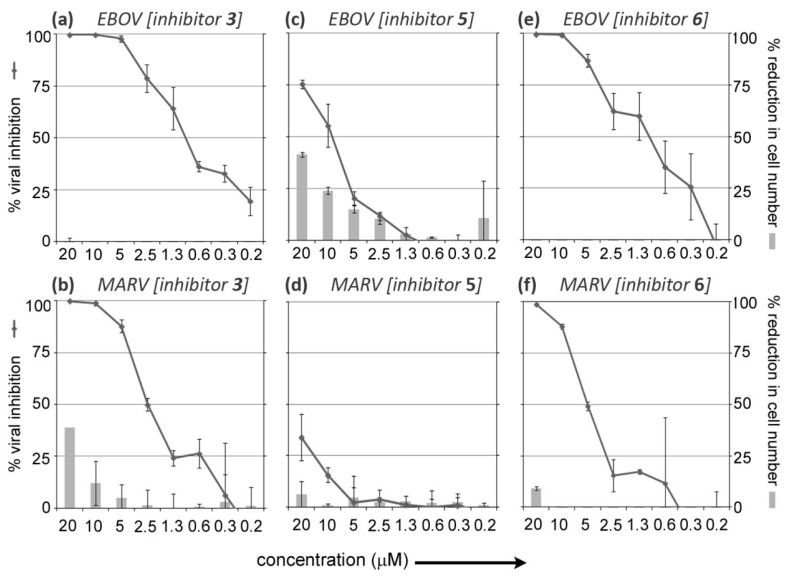
Dose**-**response curves: the inhibitory efficacies and cellular toxicities (based on reduction in cell number in compound treated and infected samples *versus* DMSO only treated and infected controls) of **3**, **5**, and **6** at eight concentrations during Ebola (EBOV; Zaire-95 isolate) and Marburg (MARV; Ci67 isolate) infection. (**a**) Dose-response curve for **3** (both cellular protection and toxicity) during EBOV infection; (**b**) Dose-response curve for **3** (both cellular protection and toxicity) during MARV infection; (**c**) Dose-response curve for **5** (both cellular protection and toxicity) during EBOV infection; (**d**) Dose-response curve for **5** (both cellular protection and toxicity) during MARV infection; (**e**) Dose-response curve for **6** (both cellular protection and toxicity) during EBOV infection; (**f**) Dose-response curve for **6** (both cellular protection and toxicity) during MARV infection.

Dose-response studies were conducted to more closely examine the efficacies and toxicities of **3** and **5** at eight concentrations during both EBOV and MARV cellular infection (Table 1, Figure 2). For **3**, the EBOV infection dose-response study indicated that the inhibitor possesses an IC_50_ = 0.696 µM ± 0.13 µM (Table 1), and as with its preliminary analysis, provides 100% (±0.0%) cellular protection at 20 µM - with no associated toxicity (Figure 2a). Hence, the protection afforded by **3** at 20 µM is approximately two-fold > than that of its parent compound (*i.e.*, **1**, which provided 53% cellular protection when previously tested at 20 µM). Moreover, **3** continued to provide approximately 100% cellular protection at both 10 and 5 µM (Figure 2a), and >50% protection at 2.5 and 1.3 µM (Figure 2a).

**Table 1:** IC_50_ values against Ebola (EBOV) and Marburg (MARV) cellular infection.

Compound	IC_50_ against EBOV	IC_50_ against MARV
**3**	0.696 µM ± 0.13 µM	2.76 µM ± 0.21 µM
**5**	12.98 µM ± 0.17 µM	ND *
**6**	1.13 µM ± 0.28 µM	10.51 µM ± 0.31 µM

* ND: not determined due to poor efficacy and associated variability and toxicity.

The dose-response study evaluating the cellular protection provided by **3** during MARV infection indicated that the inhibitor possesses an IC_50_ = 2.76 µM ± 0.21 µM (Table 1). However, at 20 µM, **3** caused 39% ± 0.0% reduction in cell number (Figure 2b). Nevertheless, when tested at 10 and 5 µM, the inhibitor continued to provide efficacious cellular protection: 99% (±1%) and 88% (±3%), respectively (Figure 2b), with markedly decreased toxicity: 12% (±11%) and 5% (±7%), at 10 and 5 µM, respectively. Interestingly, the cellular protection provided by **3** at 2.5 and 1.3 µM in the MARV assay is significantly lower *versus* the level of protection provided by the same concentrations of this inhibitor in the EBOV assay (compare Figure 2a *versus* Figure 2b).

Dose-response studies for **5**, not surprisingly (based on data from the preliminary analysis described above), indicated that this inhibitor provides significantly weaker cellular protection (*i.e.*, compared to **3**) during both EBOV and MARV infection (compare Figure 2a,b *versus* Figure 2c,d). When tested in the EBOV assay, compound **5** was found to have an IC_50_ = 12.98 µM ± 0.17 µM (Table 1), while its IC_50_ value could not be determined for the MARV infection assay due to poor efficacy and associated variability and toxicity (Figure 2d).

Furthermore, the cellular protection provided by **5** during EBOV infection was offset by cellular toxicity. For example, while providing 75% ± 2% cellular protection at 20 µM, this efficacy was offset by a 41% ± 1% reduction in cell number at the same concentration (Figure 2c).

#### 2.2.2. Comparison of the Inhibitory Efficacies and Toxicities of Salt (Cationic/Ionized) form **3** and Basic (Unionized/Non-Salt) form **6**

Due to the high degree of inhibitory efficacy (and absence of toxicity) observed for **3** (×4 HCl salt (cationic/ionized) (see Figure 1)) during both EBOV and MARV infection, a dose-response study with its basic (non-salt/unionized) form, compound **6** (Figure 1), was subsequently conducted to determine if ionization state might affect efficacy and/or toxicity.

As shown in Figure 2e,f, the dose-response results for **6** indicate that the basic (non-salt/unionized) form of the inhibitor is less efficacious than the salt (cationic/ionized) form of the inhibitor (*i.e.*, **3**) in both the EBOV and MARV assays (compare Figure 2a,b *versus* Figure 2e,f). With respect to EBOV inhibitory efficacy, compound **6** has an IC_50_ = 1.13 µM ± 0.28 µM (Table 1), which is approximately 2-fold less efficacious than that of **3**. It is speculated that salt **3 **(cationic/ionized) is more potent than its basic (non-salt/unionized) form **6**, because it possesses increased solubility in the assay. Interestingly, both **3** and **6** are equally efficacious at 20 µM and 10 µM, but at lower concentrations, the inhibitory efficacy of **6** declines more rapidly than **3** (compare Figure 2a *versus* Figure 2e), which seems to further support our hypothesis regarding the increased solubility of salt **3** in the assay (as indicated above). Nevertheless, it is notable that **6**, like **3**, is non-toxic to host cells in the EBOV assay (Figure 2e).

By comparison, differences in the MARV inhibitory efficacies provided by **6** and **3** were more pronounced (compare Figure 2b *versus* Figure 2f), with **6** (IC_50_ = 10.51 µM ± 0.31 µM (Table 1)) being approximately 4-fold less efficacious than **3**. Additionally, while **6** and **3** provided nearly equivalent inhibitory efficacies at 20 µM (*i.e.*, 99% ± 1% *versus* 100% ± 1%, respectively), the inhibitory efficacies of **6** declined more rapidly than the inhibitory efficacies of **3** at lower concentrations (compare Figure 2b *versus* Figure 2f). However, when toxicity is taken into account, it is notable that at 20 µM, **6**, which causes 9% ± 1% host cell reduction (Figure 2f), is approximately 4-fold less toxic than **3** (Figure 2b), and results in no host cell reduction at lower concentrations. Hence it may be argued, when taking into account the overall ratio of efficacy *versus* cellular toxicity (at different concentrations for the basic (non-salt/unionized) form **6** *versus* the salt (cationic/ionized) form **3**), that **6** and **3** provide approximately equivalent cellular protection during MARV infection.

### 2.3. The Future Development of DAAC-Based Anti-Filovirus Agents

Although only three new DAAC analogs were examined for anti-filovirus activity in this limited study, the biological results provide a significant amount of information that will aid in guiding the future development of this chemotype. The most important result from the study is the indication that the bis-2,8-methyl substituents of previously reported DAAC-based inhibitors of EBOV infection [14] are not required for inhibitory potency. Indeed, the results for des-methyl analogs **3** and **6** punctuate this observation. Specifically, by simply removing the bis-2,8-methyl substituents, highly efficacious inhibitors of both EBOV and MARV cellular infection were derived for a significantly less efficacious parent (*i.e.*, **1**). Furthermore, to the best of our knowledge, **3**, which possesses an IC_50_ in the sub-µM-range, is one of the most efficacious small molecule inhibitors of EBOV cellular infection reported to date.

With respect to **5**, we discovered that replacing the bis-2,8-methyl substituents of **1** with bulkier moieties (*i.e.*, the bis-2,8-amide-ethylmorpholino substituents of **5** (Figure 1)), neither results in enhanced inhibitory efficacy nor reduces toxicity. Hence, future DAAC-based inhibitor designs will not include substituents on the 2 and 8 positions of the DAAC core scaffold.

The results also indicate that the bis-4,10-alkylamino substituents of DAAC-based inhibitors play a significant role with respect to cellular toxicity. In particular, the toxicity observed for **4** *versus* that of **3** and **6** indicates that lower pKa morpholino termini on the sub-components extending off of the 4 and 10 positions of the DAAC core are favored over more readily ionizable amino termini (for example, the secondary nitrogen termini of **2** and **4**). Therefore, future medicinal chemistry research to develop DAAC-based analogs as anti-filovirus agents will focus on incorporating bis-morpholino substituents, and/or bioisosteres, on the termini of components extending off of the 4 and 10 positions of the DAAC ring.

Finally, based on the results from both the EBOV and MARV inhibitory efficacy experiments conducted with **3** and **6**, it may be surmised that the salt (cationic/ionized) forms of DAAC-based inhibitors will provide superior anti-filovirus activities without simultaneously inducing significantly unfavorable toxicities (compare Figure 2a,b *versus* Figure 2e,f). This is an important point, as the salt (cationic/ionized) forms of derivatives of the DAAC chemotype will facilitate solubility, thereby providing greater flexibility with regard to formulation and dosing during future *in vivo* studies.

## 3. Experimental Section

### 3.1. Synthesis

The syntheses of **1** and **2** were previously reported [14]. Synthetic procedures used to prepare **3**–**6**, as well as the compound’s physical properties, and structural confirmation and purity analyses, are provided as Supplementary Information. Compounds **3**–**6** were all >95% pure (see Supplementary Information).

### 3.2. Assays to Detect Anti-Filovirus Cellular Efficacy

The anti-filovirus efficacies of the small molecules were determined using high-content imaging as described previously [15], but with minor modifications. Briefly, HeLa cells (20,000 cells/well) were seeded in 96-well BD imaging plates and incubated overnight at 37 °C under 5% CO_2_. On the next day, cells were pretreated for 2 hrs with 20 µM conc. of the compounds (for primary screening) or 2-fold diluted compounds (for dose-response studies), and then infected with 3 MOI of EBOV Zaire-95 isolate or 5 MOI of MARV Ci67 isolate (note: different MOIs are used in the EBOV and MARV assays to attain an infection rate of 70%). After 2 hrs, cells were washed to remove residual filovirus, and then further incubated for 48 hrs in medium containing the compounds. Cells were then fixed for 72 hrs in 10% formalin. After washing with PBS to remove the formalin, the cells were blocked for 1 hr at r.t. with PBS containing 3% BSA, and then stained with anti-EBOV GP antibody (6D8) or anti MARV GP antibody (9G4). After 1 hr incubation with primary antibody, cells were washed and pre-incubated for 1 hr with anti-mouse Dylight 488 secondary antibody. The cells were washed with PBS and stained with HCS Cellmask Deep Red cytoplasmic/nuclear stain (Invitrogen, 5 µg/mL diluted in PBS) and nuclear Hoechst dye 33342 (Invitrogen, 1 µg/mL diluted in PBS). The plates were imaged using an Opera confocal reader (model 3842-Quadruple Excitation High Sensitivity (QEHS), Perkin Elmer, Waltham, MA 02451) [15]. Images were analyzed within the Opera environment using standard Acapella scripts. All experiments were preformed in duplicate, and repeated twice individually.

## 4. Conclusions

The data presented herein provides pertinent information that will guide the continued development of DAAC-based analogs as anti-filovirus agents. Specifically, the anti-filovirus efficacy of parent compound **1** (Figure 1), which was previously determined to afford only moderate EBOV inhibitory efficacy (53% inhibition at 20 µM), was significantly increased against both EBOV and MARV via a simple structural modification involving the removal of its bis-2,8-methyl substituents. In particular, the resulting analog, **3** (Figure 1), is one of the most efficacious, non-toxic small molecule anti-filovirus inhibitors reported to date. Additionally, the poor inhibitory efficacy and toxicity of analog **5** (Figure 1) provides further evidence that including substituents on the 2 and 8 positions of the DAAC ring system, in general, negatively impacts inhibitory efficacy. Furthermore, the high degree of toxicity associated with analog **4** (Figure 1) indicates that synthesizing analogs with non-cyclized amino termini on the sub-components extending off of the 4 and 10 positions of the DAAC ring system should be discontinued. Finally, comparison of the inhibitory efficacies and toxicities of salt **3** (cationic/ionized) and its basic (non-salt/unionized) form, analog **6** (Figure 1), indicates that the basic form of the analog provides no benefits with respect to increased efficacy and/or reduced toxicity. This information is important for future *in vivo* testing, as using salt forms of DAAC-based derivatives will allow for more flexibility with regard to dosing concentrations.

## Supplementary Files

Supplementary File 1: PDF-Document (PDF, 379 KB)
