# Supplementary material for: A Limited Structural Modification Results in a Significantly More Efficacious Diazachrysene-Based Filovirus Inhibitor
Source: Viruses. 2012 Aug 15;4(8):1279–88. doi: 10.3390/v4081279 (PMC3446762; doi:10.3390/v4081279)
Supplement: Supplementary File 1: — PDF-Document (PDF, 379 KB) [file viruses-04-01279-s002.pdf]

Viruses **2012**, *4*, 1279-1288; doi:10.3390/v4081279

OPEN ACCESS

viruses

ISSN 1999-4915

www.mdpi.com/journal/viruses

Supplementary Information

A Limited Structural Modification Results in a Significantly More Efficacious Diazachrysene-Based Filovirus Inhibitor

Života Selaković [1], Dejan Opsenica [2], Brett Eaton [3], Cary Retterer [3], Sina Bavari [3], James C. Burnett [4,*], Bogdan A. Šolaja [1,*], Rekha G. Panchal [3,*]

[1] University of Belgrade, Studentski trg 16, P.O. Box 51, 11158 Belgrade, Serbia; E-Mails: bsolaja@chem.bg.ac.rs (B.A.S.); zivota.selakovic@gmail.com (Z.S.)

[2] Institute of Chemistry, Technology, and Metallurgy, University of Belgrade, 11000 Belgrade, Serbia; E-mail: dopsen@chem.bg.ac.rs (D.O.)

[3] United States Army Medical Research Institute of Institute of Infectious Diseases, Fort Detrick, 1425 Porter Street, Frederick, MD 21702, United States; E-Mails: sina.bavari@us.arm.mil (S.B.); brett.eaton@amedd.army.mil (B.E.); rekha.panchal@us.army.mil (R.G.P.); cary.retterer@amedd.army.mil (C.R.)

[4] SAIC-Frederick, Inc., Frederick National Laboratory for Cancer Research, P.O. Box B, Frederick, MD , 21702, United States; E-Mail: burnettjames@mail.nih.gov (J.C.B.)

Table of Contents

1. **General Experimental Information** **Page S2**

2. **Synthesis and Purity of compounds 3 – 6** **Pages S2 – S7**

1. General Experimental Information

Melting points were determined on a Boetius PMHK apparatus and were not corrected. IR spectra were recorded on a Perkin-Elmer spectrophotometer FTIR 1725X. ^{1}H and ^{13}C NMR spectra were recorded on a Varian Gemini-200 spectrometer (at 200 and 50 MHz, respectively), and a Bruker Ultrashield Advance III spectrometer (at 500 and 125 MHz, respectively) in the indicated solvent (*vide infra*) using TMS as the internal standard. Chemical shifts are expressed in ppm (δ) values and coupling constants (J) in Hz. ESI–MS (HRMS) spectra of the synthesized compounds were acquired on a Agilent Technologies 1200 Series instrument equipped with Zorbax Eclipse Plus C18 (100 × 2.1 mm i.d. 1.8 μm) column and DAD detector (190-450 nm) in combination with a 6210 Time-of-Flight LC/MS instrument in positive ion mode. The samples were dissolved in pure H_2O (HPLC grade). The selected values were as follows: capillary voltage 4 kV; gas temperature 350°C; drying gas 12 L min^{-1}; nebulizer pressure 45 psig; fragmentator voltage: 70 V. Lobar LichroPrep Si 60 (40-63 μm) or LichroPrep RP-18 columns coupled to a Waters RI 401 detector were used for preparative column chromatography. Mass spectral analyses were done using electrospray ionization in positive ion mode on a Surveyor separations module coupled to a ThermoFinnigan TSQ AM triple quadrupole mass spectrometer.

1.1. HPLC purity determination

Compounds **3** - **6** were analyzed for purity (HPLC) using a Waters 1525 HPLC dual pump system equipped with an Alltech Select degasser system and dual λ 2487 UV-VIS detector. HPLC analysis was performed using two different methods:

Method A: Octadecylsilica was used as the stationary phase (Symmetry C18 analytical column, 4.6 mm × 150 mm, 5 μm, series no. 021336278136 37). Compounds were dissolved in water. Final concentrations were 0.1 - 0.5 mg/mL, and the injection volume was 10μL. Eluent was made from the following solvents: 0.2% formic acid in water (A) and methanol (B). Wavelength = 254nm.

Method B: Octadecylsilica was used as the stationary phase (Nucleosil C18 analytical column, 4 mm × 150 mm, 5 μm). Compounds were dissolved in water. Final concentrations were 0.1 - 0.5 mg/mL, and the injection volume was 10μL. Eluent was made from the following solvents: 0.2% formic acid in water (A) and methanol (B). Wavelength = 254 nm.

All compounds were > 95% pure.

2. Synthesis and Purity of Compounds 3 – 6

Scheme 1. The general synthetic procedures used to synthesize **3** – **6**, and intermediates (designated as **ZS##**).

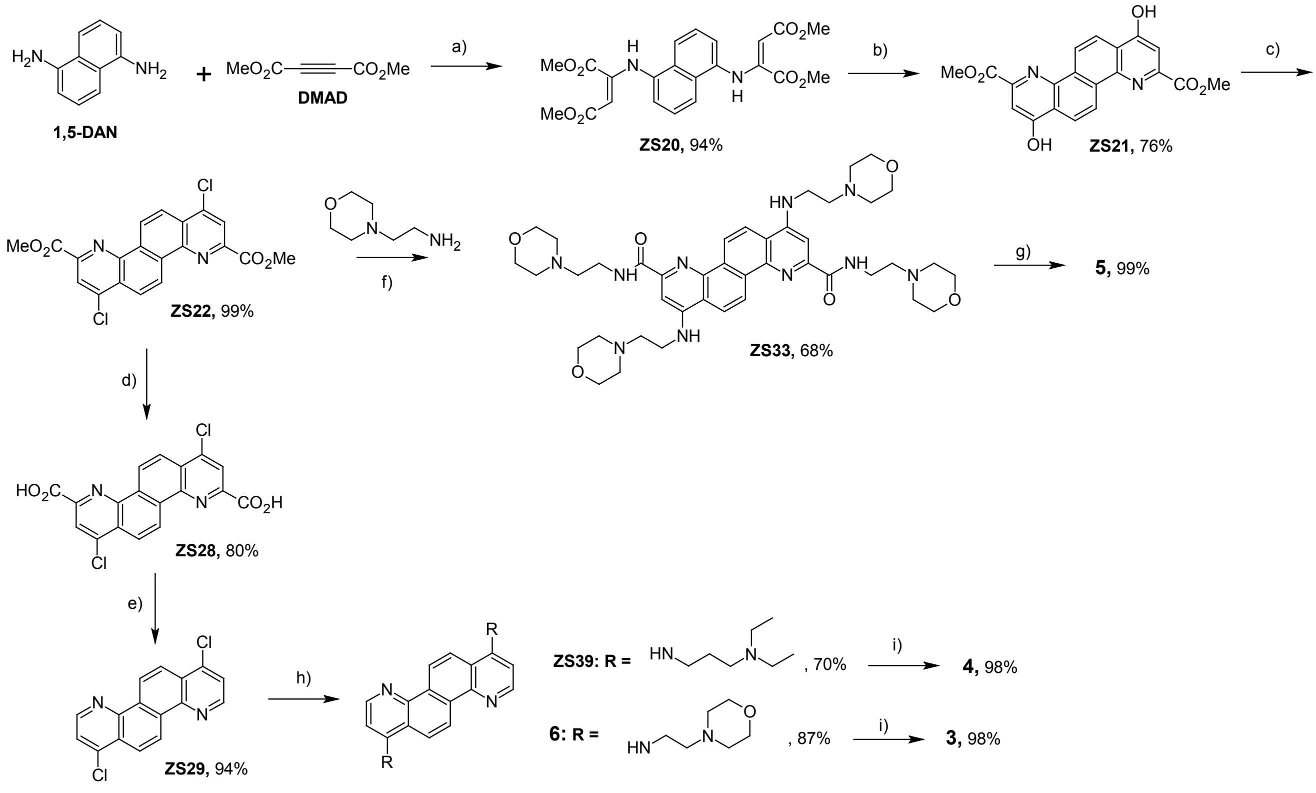

a) MeOH, 6h, r.t.; b) Ph_2O, reflux, 5 min.; c) $POCl_3$, 90 °C (6h), than 120 °C (14h); d) 1: $NaOH/H_2O$/100 °C, 24h, 2: HCl, r.t., 24h; e) Ph_2O, reflux, 10 min.; f) 210 °C, 60h, Ar; g) TFA, r.t. h) amine, Δ or MW, Ar, i) anh. MeOH/HCl

Tetramethyl (2*E*,2'*E*)-2,2'-(naphthalene-1,5-diyldiimino)bisbut-2-enedioate (ZS20).

1,5-Diaminonaphtalene (2.50 g, 15.8 mmol) was dissolved in dry methanol (40 mL), followed by the drop-wise addition of dimethyl acetylenedicarboxylate (DMAD, 4.50 mL, 5.22 g, 36.7 mmol). The reaction mixture was stirred at r.t. for 6 hrs, and then filtered. The obtained cake was washed with dry methanol. After drying under reduced pressure, 6.60 g (94 %) of **ZS20** was obtained as a yellow powder, mp = 190 - 192 °C. IR (ATR): 3241m, 3123w, 3070w, 3009w, 2957w, 1740s, 1666s, 1610s, 1515w, 1440m, 1409w, 1330w, 1268s, 1230s, 1140m, 1026m, 994w, 822w, 790m, 648m cm^{-1}. ^1H NMR (500 MHz, $CDCl_3$): 10.06 (s, 2H-N, exchangeable with D_2O), 7.95 (d, J = 8.4, H-C(8) and H-C(4)), 7.42 (t, J = 7.9, H-C(3) and H-C(7)), 6.97 (d, J = 7.4, H-C(2) and H-C(6)), 5.55 (s, 2H-C(3')), 3.78 (s, 2×$COOCH_3$), 3.60 (s, 2×$COOCH_3$). ^{13}C NMR (125 MHz, $CDCl_3$): 170.24, 164.69, 149.08, 137.07, 128.74, 125.92, 119.24, 118.64, 94.44, 52.73, 51.32. HRMS: *m*/*z* 885.28037 corresponds to

molecular formula $(C_{22}H_{22}N_2O_8)_2H^+$ (error in ppm 0.63). Microanalysis for $(C_{22}H_{22}N_2O_8 \times 0.5H_2O)$: calculated: C 58.53; H 5.14; N 6.21; found: C 58.46; H 4.67; N 6.35.

Dimethyl 1,7-dihydroxyquinolino[8,7-*h*]quinoline-3,9-dicarboxylate (ZS21).

Powdered **ZS20** was added to refluxing diphenyl ether (40 mL). The reaction mixture was stirred for 5 min and then cooled to r.t. The obtained precipitate was filtered and dried under reduced pressure. The yield was 4.22 g (76 %). **ZS21**: dark-yellow powder, mp > 280ºC. IR (ATR): 3402m, 2956w, 2896w, 2375w, 2221w, 2141w, 1721s, 1619m, 1593m, 1524s, 1468m, 1447m, 1397m, 1374s, 1286m, 1260s, 1235m, 1221m, 1156w, 1113w, 1010m, 927w, 868w, 800w, 784w, 745w, 628w, 537w cm^{-1}. ^{1}H NMR (500 MHz, TFA): 9.42 (d, J = 9.4, H-C(6) and H-C(12)), 9.08 (d, J = 9.3, H-C(5) and H-C(11)), 8.43 (s, H-C(2) and H-C(8)), 4.43 (s, 2×$COOCH_3$). ^{13}C NMR (125 MHz, TFA): 175.27, 162.34, 144.08, 139.55, 128.72, 125.60, 125.19, 124.71, 111.65, 57.63. HRMS: *m*/*z* 379.09209 corresponds to molecular formula $C_{20}H_{14}N_2O_6H^+$ (error in ppm -0.99). Microanalysis for $(C_{20}H_{14}N_2O_6)$: calculated: C 63.49; H 3.73; N 7.40; found: C 63.15; H 3.75; N 7.50.

Dimethyl 1,7-dichloroquinolino[8,7-*h*]quinoline-3,9-dicarboxylate (ZS22).

A suspension of **ZS21** (2.00 g, 5,29 mmol) in $POCl_3$ (32 mL) was heated at 90 °C for 6 hrs, followed by continued heating at 120°C for 14 hrs. Upon cooling to r.t., the mixture was slowly poured onto dry methanol (400 mL) at 5°C. The resulting precipitate was filtered and washed with methanol. The obtained dichloride, **ZS22**, was dried under reduced pressure. The yield was 2.18 g (99 %). **ZS22**: pale brown powder, mp > 280ºC. IR (ATR): 3102m, 3058w, 3007w, 2956w, 2854w, 1723s, 1582m, 1482w, 1448m, 1406w, 1365w, 1330s, 1282m, 1245m, 1204m, 1138m, 1080w, 1007w, 959w, 918w, 874w, 836w, 787w, 752w, 589w cm^{-1}. ^{1}H NMR (500 MHz, TFA): 9.73 (d, J = 9.4, H-C(6) and H-C(12)), 8.88 (d, J = 9.3, H-C(5) and H-C(11)), 8.77 (s, H-C(2) and H-C(8)), 4.23 (s, 2×$COOCH_3$). ^{13}C NMR (125 MHz, TFA): 165.38, 155.68, 145.03, 142.91, 132.58, 132.11, 129.09, 126.93, 126.75, 57.30. Microanalysis for $(C_{20}H_{12}Cl_2N_2O_4 \times H_2O)$: calculated: C 55.45; H 3.26; N 6.47; found: C 56.02; H 3.34; N 6.44.

1,7-Dichloroquinolino[8,7-*h*]quinoline-3,9-dicarboxylic acid (ZS28).

A suspension of **ZS22** (500 mg, 1.20 mmol) in NaOH (192 mg, 4.81 mmol) dissolved in water (20 mL) was refluxed for 24 hrs at 110°C. Upon cooling to r.t., the resulting sodium salt was suspended in conc. HCl (20 mL) and the mixture was stirred 24hrs under ultra-sound irradiation. The obtained solid was filtered, washed with water until to pH 7, and dried under reduced pressure. The yield was 372 mg (80 %). **ZS28**: off-white powder, mp > 280 ºC. IR (ATR): 3437w, 3100m, 2886w, 2602w, 2484w, 2359w, 1723s, 1624m, 1582s, 1478m, 1417m, 1377m, 1359m, 1287w, 1251m, 1222m, 1184w, 1156w, 1081w, 995w, 885w, 836m, 787w, 754m cm^{-1}. ^{1}H NMR (500 MHz, TFA): 9.78 (d, J = 9.3, H-C(6) and H-C(12)), 8,97 (d, J = 9.3, H-C(5) and H-C(11)), 8.92 (s, H-C(2) and H-C(8)). ^{13}C NMR (125 MHz, TFA): 166.35, 155.47, 144.84, 142.84, 132.62, 132.00, 128.92, 126.81, 126.59. Microanalysis for $(C_{18}H_8Cl_2N_2O_4)$: calculated: C 55.84; H 2.08; N 7.24; found: C 55.78; H 2.01; N 7.23.

1,7-Dichloroquinolino[8,7-h]quinoline (ZS29).

Powdered **ZS28** (330 mg, 0.85 mmol) was added to refluxing diphenyl ether (10 mL). The reaction mixture was stirred for 10 min and cooled to r.t. The obtained precipitate was filtered, washed with acetone, and dried under reduced pressure. The yield was 239 mg (94 %). **ZS29**: off-white powder, mp > 280 °C. IR (ATR): 3064w, 1578s, 1496w, 1466w, 1412m, 1361w, 1332w, 1278w, 1235w, 1192w, 1117w, 1068w, 925w, 836s, 784s, 756m, 632w, 580w cm^{-1}. ^{1}H NMR (500 MHz, TFA): 9.46 (d, *J* = 9.4, H-C(5) and H-C(11)), 9.36 (d, *J* = 6.2, H-C(3) and H-C(9)), 9.02 (d, *J* = 9.4, H-C(6) and H-C(12)), 8.50 (d, *J* = 6.2, H-C(2) and H-C(8)). ^{13}C NMR (125 MHz, TFA): 160.12, 146.80, 138.40, 131.46, 128.81, 128.28, 127.87, 126.95. HRMS: *m/z* 299.01260 corresponds to molecular formula $C_{16}H_8Cl_2N_2H^+$ (error in ppm -3.78).

***N,N′*-Bis[3-(diethylamino)propyl]quinolino[8,7-h]quinoline-1,7-diamine (ZS39).**

A suspension of dichloride **ZS29** (100 mg, 0,33 mmol) and *N,N*-diethylpropane-1,3-diamine (2.5 mL, 16 mmol) was stirred at reflux under argon for 60 hrs. The mixture was then poured onto ice-cold water (20 mL), and the solid was filtered, washed with water, and dried at 45°C under reduced pressure. The yield was 114 mg (70 %). **ZS39**: pale-gray powder, mp = 160 – 162°C. IR (ATR): 3232m, 3073w, 3027w, 2960m, 2927w, 2872w, 2824m, 1592s, 1541s, 1470w, 1432w, 1386w, 1355w, 1336w, 1296w, 1237w, 1192w, 1170w, 1134w, 1069w, 1034w, 833w, 802w, 757w cm^{-1}. ^{1}H NMR (500 MHz, TFA): 8.76 (d, *J* = 9.4, H-C(5) and H-C(11)), 8.50 (d, *J* = 7.2, H-C(3) and H-C(9)), 8.46 (d, *J* = 9.4, H-C(6) and H-C(12)), 7.08 (d, *J* = 7.4, H-C(2) and H-C(8)), 3.80 (t, *J* = 7.0, 4H-C(1’)), 3.43 – 3.40 (m, 4H-C(3’)), 3.38 – 3.24 (m, 8H-C(4’)), 2.41 – 2.35 (m, 4H-C(2’)), 1.34 (t, *J* = 7.3, 12H-C(5’)). ^{13}C NMR (125 MHz, TFA): 159.03, 144.25, 137.69, 127.70, 123.22, 122.69, 118.95, 103.19, 52.68, 50.53, 43.12, 25.29, 9.57. HRMS: m/z 487.35266 corresponds to molecular formula $C_{30}H_{42}N_6H^+$ (error in ppm -3.52). Microanalysis for ($C_{30}H_{42}N_6 \times 1.5H_2O$): calculated: C 70.14; H 8.83; N 16.36; found: C 69.61; H 8.24; N 16.58.

***N,N′*-Bis[3-(diethylamino)propyl]quinolino[8,7-h]quinoline-1,7-diamine tetrahydrochloride (4).**

ZS39 (10.0 mg, 0,0205 mmol) was suspended in 40% HCl in dry MeOH (1 mL), and the reaction mixture was vigorously stirred for 1 hr at r.t. The solvent was then removed under reduced pressure, and the remaining solid was suspended in dry EtOH (2 mL), filtered, and washed again with EtOH. Upon drying at 40°C under reduced pressure, the desired product was obtained. The yield was 12.8 mg (98 %). **4**: pale-gray powder, mp > 280 °C. IR (ATR): 3333s, 2980s, 2672m, 1622s, 1570m, 1502w, 1472w, 1440w, 1398w, 1354w, 1224w, 1144w, 1094w, 1036w, 820w, 780w, 747w cm^{-1}. ^{1}H NMR (500 MHz, D_2O): 8.55 – 8.52 (m, H-C(3) and H-C(5) and H-C(9) and H-C(11)), 8.36 (d, *J* = 9.3, H-C(6) and H-C(12)), 7.18 (d, *J* = 7.2, H-C(2) and H-C(8)), 3.84 (t, *J* = 7.0, 4H-C(1’)), 3.41 – 3.37 (m, 4H-C(3’)), 3.30 (q, *J* = 7.3, 8H-C(4’)), 2.33 – 2.27 (m, 4H-C(2’)), 1.33 (t, *J* = 7.3, 12H-C(5’)). ^{13}C NMR (125 MHz, D_2O): 155.87, 141.70, 134.86, 124.63, 120.80, 119.99, 115.89, 100.79, 49.05, 47.50, 40.54, 22.34, 8.21. HRMS: m/z 487.35583 corresponds to molecular formula $C_{30}H_{42}N_6H^+$ (error in ppm 3.00); 244.18082 corresponds to molecular formula $C_{30}H_{42}N_6H_2^{2+}$ (error in ppm -0.02).

Microanalysis for ($C_{30}H_{46}C_{14}N_6{\times}8H_2O$): calculated: C 46.39; H 8.05; N 10.82; found: C 46.38; H 8.05; N 10.82. HPLC purity: method A, using gradient protocol 0 - 3 min 99% A, 3 - 9 min 99%→60% A, 9 - 11 min 60 % A, 11 - 12 min 60% → 99% A, flow 1.0 mL/min, RT 6.102, area 95.93%; method B, using gradient protocol 0 - 3 min 99% A, 3 - 9 min 99%→60% A, 9 - 11 min 60 % A, 11 - 12 min 60% → 99% A, flow 1.0 mL/min, RT 8.580, area 95.91%.

***N,N′*-bis[2-(morpholin-4-yl)ethyl]quinolino[8,7-*h*]quinoline-1,7-diamine (6)**.

ZS29 (35.4 mg, 0,12 mmol) and 2-(morpholin-4-yl)ethanamine (160 mg, 1.23 mmol) were dissolved in NMP (1 mL) in a MW cuvette under argon. The reaction mixture was subjected to MW irradiation using a *Biotage Initiator 2.5* apparatus for 6 hrs at 18 °C. The cooled reaction mixture was poured onto ice-water (20 mL). The obtained precipitate was filtered, washed with water, and dried under reduced pressure. The yield was 50.10 mg (87 %). **6**: pale-brown powder, mp > 280°C. IR (ATR): 3436m, 3413w, 2962w, 2855m, 2813m, 1594s, 1534s, 1505w, 1456m, 1332w, 1275w, 1239w, 1178w, 1142m, 1118s, 1070w, 1030w, 952w, 911w, 862w, 822 w, 763w. ^{1}H NMR (500 MHz, TFA): 8.97 (d, J = 9.4, H-C(5) and H-C(11)), 8.68 (d, J = 7.2, H-C(3) and H-C(9)), 8.58 (d, J = 9.4, H-C(6) and H-C(12)), 7.30 (d, J = 7.2, H-C(2) and H-C(8)), 4.40 – 4.35 (m, 8H-C(4’)), 4.11 (t, J = 12.4, 4H-C(1’)), 3.98 – 3.91 (m, 8H-C(3’)), 3.57 – 3.48 (m, 4H-C(2’)). ^{13}C NMR (125 MHz, TFA): 159.11, 144.79, 137.79, 127.84, 123.35, 123.27, 119.30, 103.69, 66.14, 57.79, 55.27, 40.13. HRMS: *m*/*z* 487.28029 corresponds to molecular formula $C_{28}H_{34}N_6O_2H^+$ (error in ppm -2.69).

***N,N′*-bis[2-(morpholin-4-yl)ethyl]quinolino[8,7-h]quinoline-1,7-diamine tetrahydrochloride (3).**

Crude base **6** (10.0 mg, 0,021 mmol) was suspended in 40% HCl in dry MeOH (1 mL), and the reaction mixture was vigorously stirred for 1 hr at r.t. The solvent was then removed under reduced pressure, and the remaining solid was suspended in dry EtOH (2 mL), filtered, and washed again with EtOH. Upon drying at 40 °C under reduced pressure, the desired product was obtained. The yield was 12.7 mg (98 %). **3**: light brown powder, mp > 290°C. ^{1}H NMR (500 MHz, D_2O): 8.41 (d, J = 6.4, H-C(3) and H-C(9)), 8.09 (d, J = 9.0, H-C(5) and H-C(11)), 8.00 (d, J = 8.7, H-C(6) and H-C(12)), 7.14 (d, J = 6.5, H-C(2) and H-C(8)), 4.19 (bs, 4H-C(1’)), 4.10 (bs, 8H-C(4’)), 3.76 (bs, 4H-C(2’)), 3.62 (bs, 8H-C(3’)). ^{13}C NMR (125 MHz, D_2O): 155.36, 142.11, 134.09, 123.68, 120.94, 119.36, 115.61, 101.36, 63.70, 54.04, 52.22, 37.57. HRMS: *m*/*z* 487.28080 corresponds to molecular formula $C_{28}H_{34}N_6O_2H^+$ (error in ppm -1.65). HPLC purity: method A, using gradient protocol 0 - 2min 44% A →42%A, 2 - 6 min 42%→40% A, 6 - 8 min 40%→ 30 % A, 8 - 9 min 30% → 44% A, flow 0.5 mL/min, RT 2.084, area 96.93 %; method B, using gradient protocol 0 - 2 min 70%→68% A, 2 - 6 min 68%→64% A, 6 - 8 min 64 % A, 8 - 10 min 64% → 50% A, 10 - 11 min 50% → 70% A, flow 0.5 mL/min, RT 2.069, area 96.66 %.

N,N'-bis[2-(morpholin-4-yl)ethyl]-1,7-bis{[2-(morpholin-4-yl)ethyl]amino}quinolino[8,7-h]quinoline-3,9-dicarboxamide (ZS33).

ZS22 (200 mg, 0.48 mmol) and 2-(morpholin-4-yl)ethanamine (5 mL, 38 mmol) were mixed under argon with vigorous stirring, and the reaction mixture was heated for 60 hrs at 210ºC. The reaction mixture was subsequently cooled and poured onto ice-water (40 mL). The obtained precipitate was filtered, washed with water and acetone, and dried under reduced pressure. The yield was 264 mg (68 %). **ZS33**: pale yellow-green powder, mp = 264 - 266ºC. IR (ATR): 3358m, 2952w, 2880w, 2847w, 2816m, 1674s, 1592m, 1524s, 1467w, 1338w, 1297w, 1274w, 1170w, 1140w, 1116s, 1030w, 996w, 945w, 915w, 856w, 804w, 748w, 670w. ^{1}H NMR (500 MHz, TFA): 9.02 (d, J = 9.4 Hz, H-C(6) and H-C(12)), 8.58 (d, J = 9.4, H-C(5) and H-C(11)), 7.92 (s, H-C(2), H-C(8)), 4.40 – 4.37 (m, 4H-C(1')), 4.34 – 4.26 (m, 4Heq-C(4') and 4Heq-C(4'')), 4.13 – 4.10 (m, 4H-C(1'')), 4.08 – 4.00 (m, 4Hax-C(4') and 4Hax-C(4'')), 3.91 – 3.82 (m, 4H-C(2'), 4Heq-C(3') and 4Heq-C(3''))), 3.67 – 3.69 (m, 4H-C(2'')), 3.47 – 3.37 (m, 4Hax-C(3') and 4Hax-C(3'')). ^{13}C NMR (125 MHz, TFA): 160.30, 143.52, 137.45, 128.26, 124.64, 123.43, 119.92, 117.65, 102.05, 66.22, 66.05, 59.63, 57.27, 55.47, 55.26, 40.75, 37.82. HRMS: m/z 799.46053 corresponds to molecular formula $C_{42}H_{58}N_{10}O_6H^+$ (error in ppm -1.03); 400.23404 corresponds to molecular formula $C_{42}H_{58}N_{10}O_6H_2^{2+}$ (error in ppm -0.68). Microanalysis for ($C_{42}H_{58}N_{10}O_6 \times 1.5H_2O$): calculated: C 61.07; H 7.44; N 16.96; found: C 61.05; H 7.00; N 17.34.

1,7-bis{[2-(morpholin-4-ium-4-yl)ethyl]amino}-3,9-bis{[2-(morpholin-4-ium-4-yl)ethyl]carbamoyl}quinolino[8,7-h]quinolinediium hexakis(trifluoroacetate) (5).

Crude base **ZS33** (10 mg, 0,013 mmol) was dissolved in TFA (1 mL) and stirred for 1 hr. The solvent was then removed under reduced pressure, and the remaining solid was suspended in dry EtOH (2 mL), filtered, and washed again EtOH. Upon drying at 40°C under reduced pressure, the desired product was obtained. The yield was 18.4 mg (99 %). **5**: brown powder, mp > 280ºC. IR (ATR): 3326m, 3017w, 2628w, 2360w, 1676s, 1600m, 1535m, 1457w, 1372w, 1312w, 1268w, 1201s, 1132s, 835w, 800w, 721w cm^{-1}. ^{1}H NMR (500 MHz, CD_3OD): 9.52 (d, J = 9.2 Hz, H-C(6) and H-C(12)), 8.37 (d, J = 9.2, H-C(5) and H-C(11)), 7.58 (s, H-C(2) and H-C(8)). ^{13}C NMR (125 MHz, CD_3OD): 169.42, 152.98, 150.13, 146.09, 133.59, 124.90, 120.51, 119.56, 99.33, 65.25, 65.19, 58.65, 56.55, 53.79, 53.76, 38.60, 35.50. HRMS: m/z 799.46207 corresponds to molecular formula $C_{42}H_{58}N_{10}O_6H^+$ (error in ppm 0.90). Microanalysis for ($C_{54}H_{64}F_{18}N_{10}O_{18} \times 2H_2O$): calculated: C 42.69; H 4.51; N 9.22; found: C 42.49; H 4.59; N 9.84. HPLC purity: method A, gradient protocol 0 - 3 min 82% A, 3 - 9 min 82%→18% A, 9 - 11 min 18 % A, 11 - 12 min 18% → 82% A, flow 0.5 mL/min., RT 3.531, area 95.34%; method B, gradient protocol 0 - 3 min 80% A, 3 - 9 min 80%→20% A, 9 - 11 min 20 % A, 11 - 12 min 20%→80% A, flow 0.5 mL/min., RT 8.159, area 96.44%.
